# Varicella-zoster virus reactivation causing herpes zoster ophthalmicus (HZO) after SARS-CoV-2 vaccination – report of three cases

**DOI:** 10.1186/s12348-021-00260-4

**Published:** 2021-09-16

**Authors:** Ioannis Papasavvas, Christian de Courten, Carl P. Herbort

**Affiliations:** 1 Retinal and Inflammatory Eye Diseases, Centre for Ophthalmic Specialised Care (COS), Clinic Montchoisi Teaching Centre, Lausanne, Switzerland; 2 Clinic Montchoisi Teaching Centre, Lausanne, Switzerland

**Keywords:** Herpes-zoster ophthalmicus, SARS-CoV-2 vaccine, mRNA vaccine

## Abstract

**Purpose:**

We are reporting 3 patients who presented acute zoster ophthalmicus (HZO), an activation of varicella-zoster virus, after mRNA anti-SARS-CoV-2 vaccination, seen directly or referred to our center.

**Cases:**

A 73-year-old woman with history of ocular sarcoidosis presented HZO in the right V1 dermatome 16 days after a single booster dose of vaccination (Pfizer BioNTech). A 69-year-old woman presented HZO in her V1 left dermatome, occurring 10 days after her first dose of Pfizer BioNTech vaccine. A 72-year-old woman with no history of autoimmune pathology, candidate for cataract surgery, presented 13 days after the first dose of a Moderna mRNA vaccine with an eruption in the left V1 dermatome. All patients presented the VZV infection after their first dose of a mRNA type of vaccine. Treatment with Valacyclovir 1000 mg × 3/ day for 7–14 days was efficient in all cases.

**Conclusion:**

Vaccines have been reported in the past to trigger different types of side effects such as viral or flu-like symptoms. It is only logical to note many different side effects for SARS-CoV-2 vaccines as the population vaccinated is exceeding any other number in history. VZV is one of the more severe side effects that can, however, be treated. It is quite obvious that, as far as mRNA vaccines are concerned, and probably also other anti-SARS-CoV-2 vaccines, that the benefit of vaccination certainly outweighs the possible but very low risk of ocular side effects that can mostly be treated.

## Introduction

Since 2019, the Covid-19 pandemic has changed the world and the life of everyone, ophthalmologists included. One of the first whistle-blower, moreover, was an ophthalmologist who later died of the disease. The whole scientific community was focussed on the rapid development of a vaccine which, once available, resulted in a vaccination campaign of an unprecedented scale. Adverse events, including ocular side-effects, had to be anticipated following such a massive number of vaccinations. In Switzerland vaccination was relatively promptly open to all age classes.

Herpes zoster ophthalmicus (HZO) of the V1 trigeminal nerve branch can be at the origin of severe skin and ocular involvement including kerato-uveitis [1]. Since the mid-1980s, with the availability of antivirals such as acyclovir, HZO severe ocular complications were reduced from 21% to 4% [2]. Adequate treatment with oral acyclovir [3] or more recently with valacyclovir allows to control the disease with minimal consequences except for zoster related neurogenic pain in some cases.

The development of HZO (shingles) has been reported as an adverse event following vaccination with mRNA vaccines in patients with autoimmune rheumatic diseases [4]. We report three cases seen in our centre, specialised in uveitis and intraocular inflammatory diseases, who developed a V1 trigeminal varicella-zoster infection after having received an mRNA SARS-CoV-2 vaccination.

## Cases

### Case: 1

**Fig. 1:**
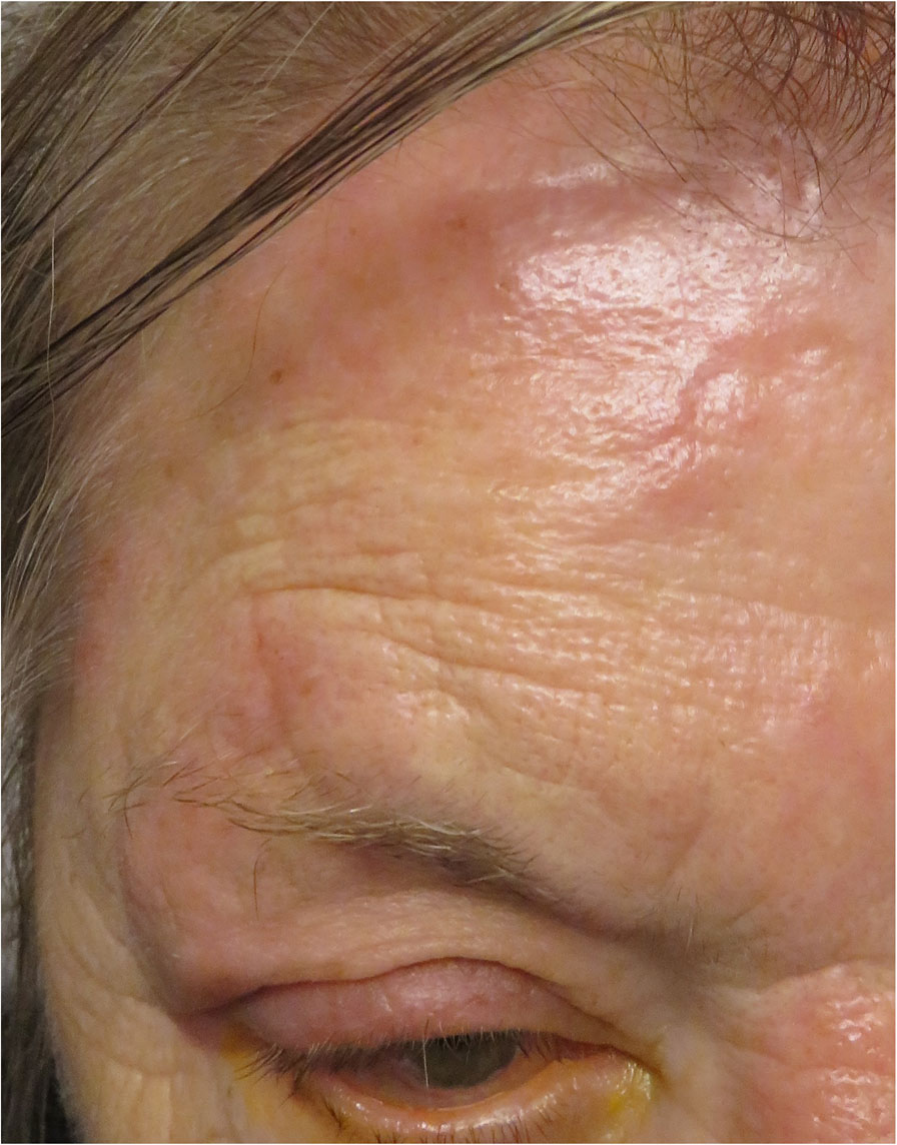
Right herpes zoster ophthalmicus. Presence of a typical HZO eruption in the right V1 trigeminal dermatome in patient 1

The first patient was a 73-year-old woman with a history of ocular sarcoidosis. She presented HZO in the right V1 dermatome (Fig. 1) 16 days after a single booster dose of vaccination (Pfizer BioNTech), 8 months after having suffered from covid-19 infection. She was referred by her dermatologist 4 days after he started a valacyclovir treatment (1000 mg 3x/ day). BCVA was 0.3 OD and 1.0 OS. At the slit lamp examination, the cornea was clear, without signs of dendrite formation and anterior chamber was normal. Laser flare photometry (LFP) showed subclinical inflammation due to ocular sarcoidosis with values of 62.9 ph/ms OD et 40.9 ph/ms OS (normal 4–6 ph/ms). Intraocular pressure (IOP) was 10 mmHg OD and 12 mmHg OG. Vitreous showed trace cells in both eyes and several quiet scars in both fundi. The patient presented extreme pain in the region of the V1 dermatome which was not controlled with 1st step analgesic drugs. We therefore prescribed capsaicin ointment (0.025%) which was very effective in relieving her pain. Evolution was rapidly favourable.

### Case: 2

**Fig. 2:**
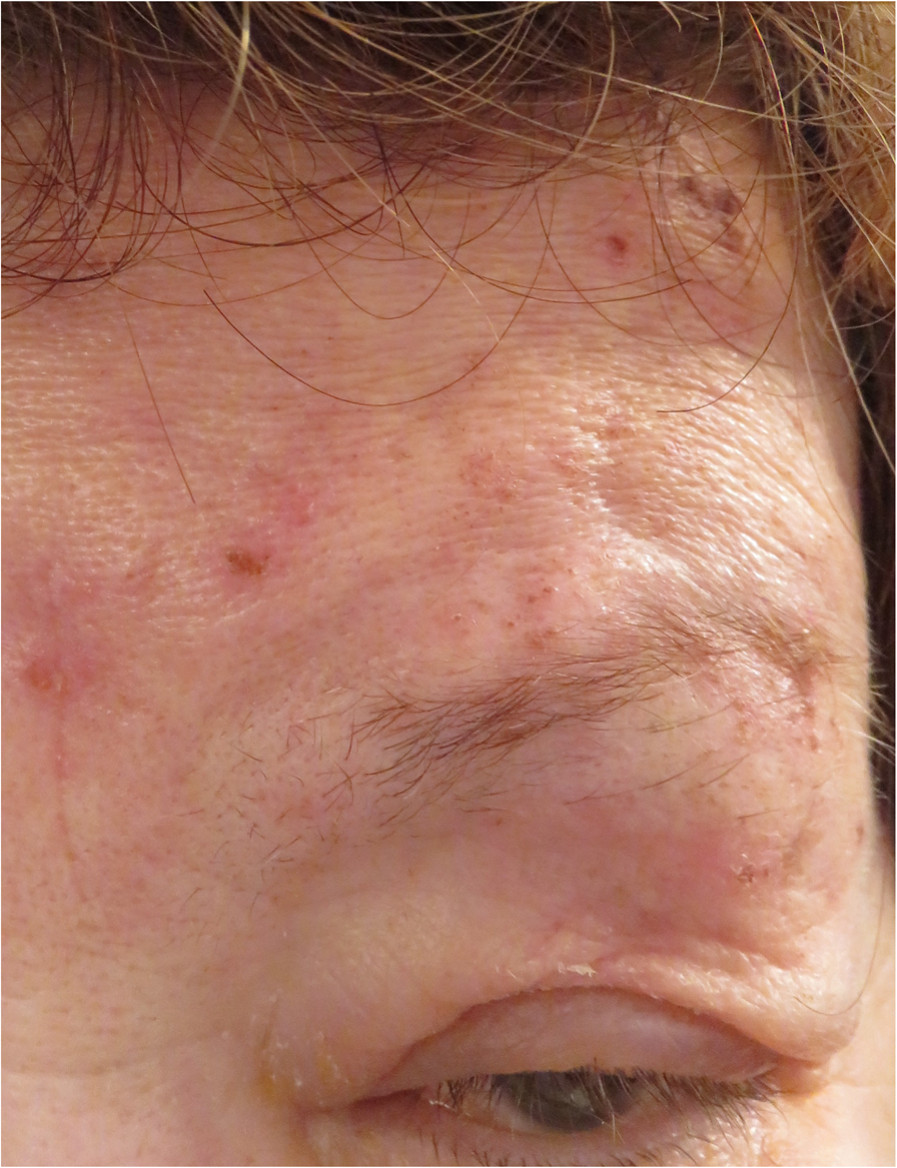
Left herpes zoster ophthalmicus. The second patient with a skin eruption in the left V1 trigeminal dermatome

**Fig. 3:**
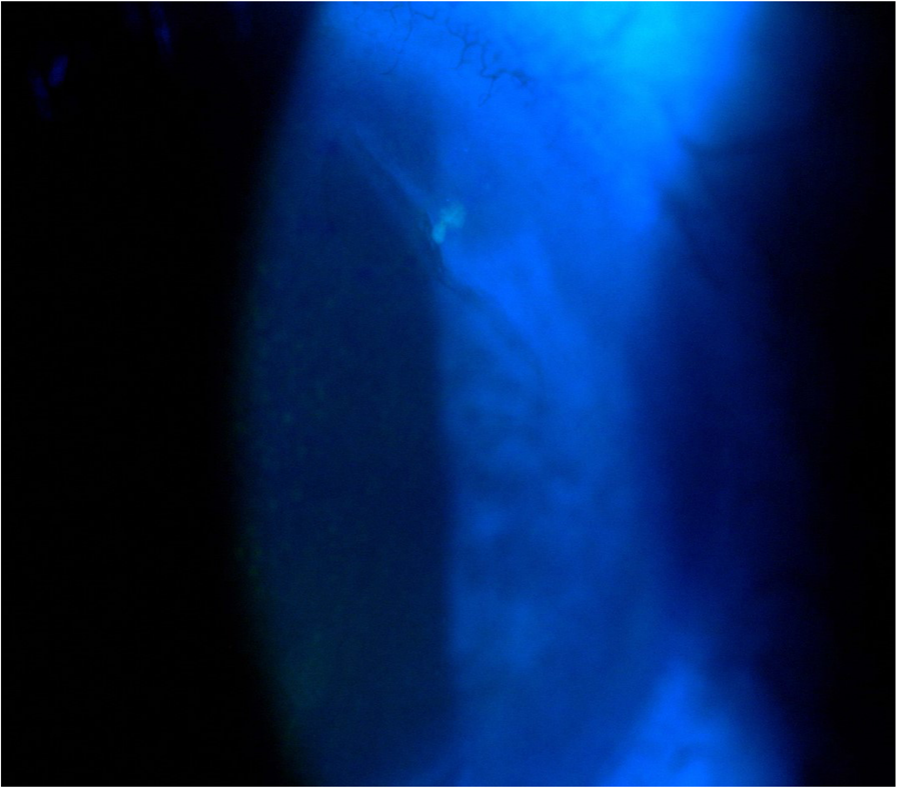
Presence of a fluorescein-positive microdendrite in the supero-nasal cornea, as typically seen in herpes zoster ophthalmicus

Our second patient, a 69-year-old woman presented HZO in her V1 left dermatome (Fig. 2), occurring 10 days after her first dose of Pfizer BioNTech vaccine. Her right eye had suffered a trauma in the past reducing her vision. At presentation she had a BCVA of 0.16 OD and 1.25 OS. Slit lamp examination showed a small dendrite in the supero-temporal cornea (Fig. 3). Anterior chamber was normal and fundoscopy was normal as well OS. LFP was 8.9 ph/ms OD and 9.3 ph/ms OS. IOP was 14 mmHg in both eyes. We started immediately a treatment of valacyclovir 1000 mg × 3/ day and acyclovir ointment × 1/day in her left eye. The dendrite disappeared without sequels after 1 week. We maintained oral valacyclovir for a total of 2 weeks because of massive skin involvement. The patient presented excruciating pain in the area of dermatome V1 not controlled by Paracetamol and Pregabalin. We also prescribed capsaicin ointment (0.025%) which relieved her pain substantially. Evolution was slowly favourable. The second dose of vaccination was postponed.

### Case: 3

**Fig. 4:**
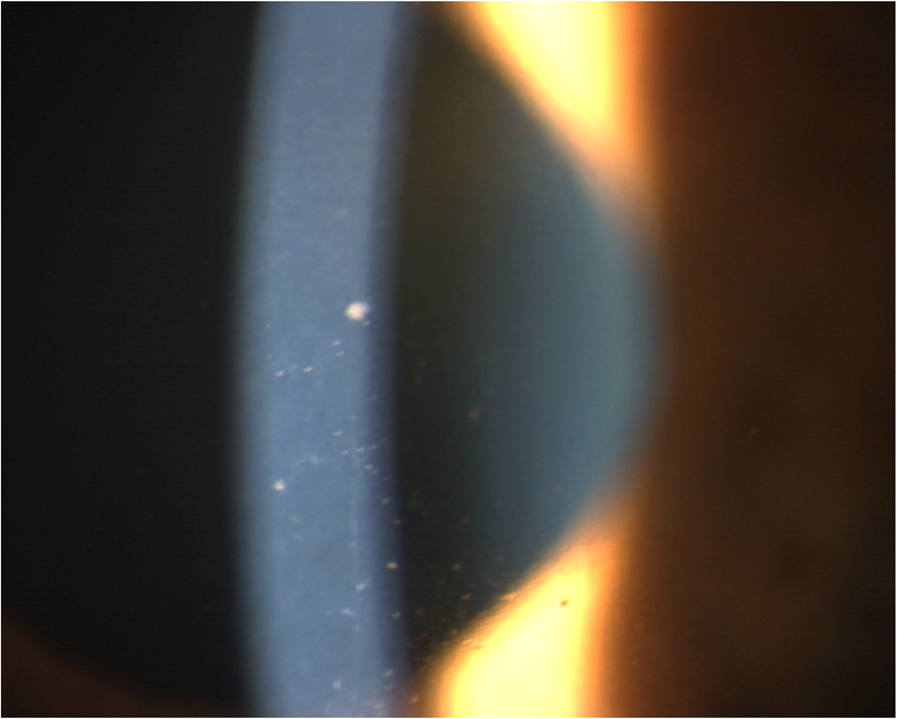
Granulomatous herpes zoster ophthalmicus uveitis. Endothelial granulomatous precipitates (KPs) of different sizes

The third patient, a 72-year-old woman planned for cataract surgery, presented 13 days after the first dose of a Moderna mRNA vaccine with an eruption in the left V1 dermatome. Her BCVA was 0.7 OD and 0.5 OS due moderate cataracts ODS. IOP was 14 mmHg both eyes. At the slit lamp there was a left conjunctival chemosis but the cornea and anterior chamber were normal. She was treated with valacyclovir 1000 3x/daily for 7 days, acyclovir ointment 3x daily, a combined antibiotic and dexamethasone 0.1% eye drop 3x daily. 10 days later the anterior chamber presented a uveitis with flare, cells and keratic precipitates and moderate Descemet folds (Fig. 4). IOP increased to 18 mmHg OS. She also complained of severe pain in the V1 dermatome and was prescribed capsaicin ointment 0.025% 3x daily. Evolution was slowly favourable with resolution of the uveitis and the skin lesions. The second dose of vaccination was postponed as well her cataract surgery.

All 3 patients had non-complicated evolution with a complete skin and ocular recovery after a mean follow-up of 105 ± 10 days.

## Discussion

Since efficacious and well tolerated systemic antiviral therapy has become available, HZO, a disease with high and severe morbidity in the past, has evolved to a disease that can successfully be managed devoid of severe complications [2]. Of interest in our 3 patients is the fact that all 3 presented unusually severe pain in the dermatome involved despite antiviral treatment having been introduced within 48–72 h. Capsaicin ointment has already found its role in diabetic neuropathy [5] and in other neuropathic pain situations [6] and has also been reported in post-zoster neuralgia [7], a therapeutic possibility not very well known among practitioners. Capsaicin is depolarizing peptidergic neurons which transmit the sensation of pain and so prevents their function. Our patients presented acute and persistent neurological pain resistant to systemic pain medications during the vaccine-induced episode of HZO and all three mentioned substantial relief of pain after the application of the capsaicin ointment.

Other virus reactivations reported after SARS-Cov-2 vaccination including Epstein-Barr virus and we noted a personal case of reactivation of herpes uveitis [8]. It is not our competence to formulate hypotheses on the mechanisms of reactivation.

Most post-mRNA-vaccination side-effects are benign and, in case of more severe side-effects, like HZO, treatment is readily available and efficient today. It is nevertheless necessary to alert the medical community of the possibility of HZO after covid vaccination. Indeed, mRNA vaccines and probably also other anti-SARS-CoV-2 vaccines are able to disturb the immunological control of the commensal herpes viruses and contribute to their resurgence. However, with the availability of effective antivirals, the benefit of vaccination largely outweighs the possible but low risk of such treatable ocular side effects.

## Data Availability

For data, please refer to corresponding author.

## Abbreviations

HZO: Herpes zoster ophthalmicus
LFP: Laser flare photometry
IOP: Intraocular pressure
